# The association between biomarkers of acrylamide and cancer mortality in U.S. adult population: Evidence from NHANES 2003-2014

**DOI:** 10.3389/fonc.2022.970021

**Published:** 2022-09-28

**Authors:** Wenbo Gu, Jiacheng Zhang, Chunling Ren, Yang Gao, Tongfang Zhang, Yujia Long, Wei Wei, Shaoying Hou, Changhao Sun, Changhong Wang, Wenbo Jiang, Junfei Zhao

**Affiliations:** ^1^ Department of Nutrition and Food Hygiene, The National Key Discipline, School of Public Health, Harbin Medical University, Harbin, China; ^2^ Comprehensive Test Center of Chinese Academy of Inspection and Quarantine, Gao Bei Dian North Rd A3, Chao Yang District, Beijing, China; ^3^ Department of Thoracic Surgery, Harbin Medical University Cancer Hospital, Harbin, China; ^4^ Department of Cardiovascular Surgery, Guangdong Cardiovascular Institute, Guangdong Provincial People’s Hospital, Guangdong Academy of Medical Sciences, Guangdong, China

**Keywords:** acrylamide, cancer mortality, inflammation, HbAA, HbGA, INFLA-score

## Abstract

The association between acrylamide (AA) and the development of cancer has been extensively discussed but the results remained controversial, especially in population studies. Large prospective epidemiological studies on the relationship of AA exposure with cancer mortality were still lacking. Therefore, we aimed to assess the association between AA biomarkers and cancer mortality in adult population from National Health and Nutrition Examination Survey (NHANES) 2003-2014. We followed 3717 participants for an average of 10.3 years. Cox regression models with multivariable adjustments were performed to determine the relationship of acrylamide hemoglobin adduct (HbAA) and glycidamide hemoglobin adduct (HbGA) with cancer mortality. Mediation analysis was conducted to demonstrate the mediated role of low-grade inflammation score (INFLA-score) in this correlation. Compared with the lowest quintile, participants with the highest quintile of HbAA, HbGA and HbAA+HbGA had increased cancer mortality risk, and the hazard ratios(HRs) were 2.07 (95%CI:1.04-4.14) for HbAA, 2.39 (95%CI:1.29-4.43) for HbGA and 2.48 (95%CI:1.28-4.80) for HbAA+HbGA, respectively. And there was a considerable non-linearity association between HbAA and cancer mortality (*p*_for non-linearity_ = 0.0139). We further found that increased INFLA-score significantly mediated 71.67% in the effect of HbGA exposure on increased cancer mortality risk. This study demonstrates that hemoglobin biomarkers of AA are positively associated with cancer mortality in adult American population and INFLA-score plays a mediated role in this process. Our findings can raise public awareness of environmental and dietary exposure to acrylamide and remind people to refrain from smoking or having acrylamide-rich foods.

## Introduction

Acrylamide (AA), a well-documented animal carcinogen and human neurotoxic compound, ubiquitously presents in industrial wastewater, production of daily cosmetics, textiles, plastics, cigarette mainstream smoke and baking and fried star chic foods (1–4). After AA is absorbed by the body, some of AA is converted into glycidamide (GA) by cytochrome P450 (5). Further, AA and GA can bind hemoglobin (Hb) to form blood adducts (6). As well-established internal biomarkers, HbAA and HbGA can provide timely and accurate information about the amounts of AA in human body for the past three months (7). AA has already been identified as 2A-class carcinogen in 2020 and the safe limits in food are determined. Therefore, the World Health Organization has called for more researches on the health hazards of AA and issued an international health alert on its health risks (8).

Animal studies showed that AA exposure could lead to scrotal mesothelial tumor in male mice (9) and could cause an elevated incidence of tumors of the central nervous system (CNS), mammary gland, ovarian and thyroid gland in female mice (10–12). Whereas in epidemiologic researches, the results for the effects of AA on cancer were not consistent. A cross-section study indicated that a 10 times increase of HbAA level was related to a 90% increase of breast cancer risk (13). And in a Netherlands cohort study, increased dietary AA intakes were associated to higher ovarian and endometrial cancer risks in postmenopausal women (14). Additionally, there were also other studies demonstrating that no correlation existed between intakes of dietary AA and increased risks of lung, liver, breast or gastrointestinal cancer in Japanese population (15–18). Therefore, a reliable cohort study is needed to provide direct evidence for the effects of AA exposure on cancer mortality in general population.

Numerous studies have illustrated the relationship between inflammation and cancer (19–21). Low-grade inflammation score (INFLA-score), composed of C-reactive protein (CRP) concentration, white blood cell (WBC) counts, platelet counts and ratio of granulocyte/lymphocyte (G/L), has been widely used in the Moli-sani studies to reflect low grade inflammatory condition (22, 23). Moreover, a recent epidemiological study indicated that AA exposure in daily life was positively associated with systemic inflammation in general population (24). Accordingly, in our study, we intended to explore whether high levels of serum AA biomarkers had an effect on cancer mortality through mediated role of inflammation, using the population in NHANES 2003-2014.

## Materials and methods

### Study population

NHANES is a stratified, multilevel study using a national population sample in the United States. The details for NHANES have been documented elsewhere (25). After excluding participants with missing information of serum AA metabolites, cancer mortality or other covariates, a total of 3717 participants aged over 18 years old with data of interviews and examinations were included in our study. The NHANES program was approved by the National Center for Health Statistics and inform consents were signed by the participants.

### Main exposure and outcome

The main exposures in our study were HbAA, HbGA and HbAA+HbGA. Besides, the main outcome was mortality status ascertained by the National Death Index (NDI) records up to the end of 2015. Cause-specific death was determined using International Classification of Diseases, Tenth Revision (ICD-10) and cancer mortality was defined as ICD-10 codes (C00-C97). Finally, a total of 513 deaths, including 118 died from cancer, were recorded for further analysis.

### Measurements of HbAA and HbGA

The levels of HbAA and HbGA in the whole blood were measured in 2003 or 2005 for one time using high-performance liquid chromatography/tandem mass spectrometry (HPLC-MS/MS) as Vesper, et al. reported (26). Briefly, 350 μl of human whole blood was treated with Edman reagent. Then AA and GA could be isolated from Hb chain and were conducted with HPLC-MS/MS. The threshold of the detection was 3 pmol/g Hb for HbAA and 4 pmol/g Hb for HbGA. Sample weights for measuring HbAA and HbGA are available in the NHANES Analytic Guidelines and the on-line NHANES Tutorial at https://wwwn.cdc.gov/Nchs/Nhanes/2003-2004/L06AGE_C.htm and https://wwwn.cdc.gov/Nchs/Nhanes/2005-2006/AMDGYD_D.htm.

### Covariates assessment

Potential covariates included age, sex (male/female), race (Mexican American, other Hispanic, non-Hispanic white, non-Hispanic black or other races), regular exercise (yes/no, defined as having moderate to high-intensity physical exercise in the past month), body mass index (BMI), education (< 9th Grade, 9-11th Grade, High School Grade, GED or Equivalent, Some College or AA degree, College Graduate or above), income (< $20,000, $20,000-$45,000, $45,000-$75,000, $75,000-$100,000 or > $100,000), current smoking (yes/no, defined as smoking in the past five days), current drinker (yes/no, defined as drinking at least 12 times in the past year), energy (kcal/d), cotinine (ng/ml), diabetes (yes/no), hypertension (yes/no), dyslipidemia (yes/no) and INFLA-score.

### Statistical analyses

The mean levels of AA hemoglobin biomarkers were displayed as mean (standard deviation) according to various demographic characteristics of participants. General linear models were applied to compare characteristics of baseline. Statistical analyses were performed using R 4.1.2 software, all the tests were two-tailed and *P* < 0.05 was regarded as statistically significant difference.

Hazard ratios (HRs) with 95% confidence intervals (CIs) were calculated using cox proportional hazards (CPH) models. The follow-up time was defined by numbers of follow-up person-months from interview date until death or end of 2015. The hemoglobin adducts of AA were classified into quintiles, with the lowest quintile considered as reference group. Then we tested four models using stepwise method with adjustment of multiple covariates. Model 1 adjusted age, sex, and race; Model 2 additionally adjusted smoking and drinking status, education level, income, BMI, regular exercise, energy and cotinine. Model 3 further adjusted disease status of diabetes, hypertension and dyslipidemia. Model 4 finally adjusted INFLA-score. Furthermore, the dose-dependent relationship between hemoglobin biomarkers of AA and cancer mortality was graphically characterized using restricted cubic spline (RCS) models.

### Sensitivity analyses

Three sets of sensitivity analyses were performed in the study. In the first set, participants with follow-up time less than 2 years (including deaths within 2 follow-up years) were excluded to evaluate whether severe disease such as cardiovascular disease could affect the results. In the second set, sub-group analysis in the non-cancer population was conducted to exclude the effect of cancer disease on cancer mortality induced by AA. In the third set, we examined whether behavior of smoking or status of diseases such as hypertension, dyslipidemia and diabetes would interact with the effect of AA exposure on cancer mortality.

## Results

### Participants characteristics

The participants of the study consisted of 49.3% male and 50.7% female, and the mean levels of AA biomarkers for them were 85.36 pmol/g Hb and 68.54 pmol/g Hb for HbAA, 65.36 pmol/g Hb and 63.17 pmol/g Hb for HbGA and 150.72 pmol/g Hb and 131.70 pmol/g Hb for HbAA+HbGA, respectively. Levels of HbAA differed significantly among different races (*P*_for trend_ = 0.003). Large amounts of HbAA, HbGA and HbAA+HbGA were mainly observed in participants with younger age, lower educational attainment, lower family income, higher energy intakes, higher cotinine and higher INFLA-score. Other details for mean levels of AA biomarkers categorized by participants characteristics were shown in **Table 1**.

**Table 1:** The levels of hemoglobin biomarkers of AA categorized by participants characteristics.

Characteristics	No.	HbAA (pmol/g Hb)		HbGA (pmol/g Hb)		HbAA +HbGA(pmol/g Hb)	
	(weighted %)	Mean	*p*_for trend_	Mean	*p*_for trend_	Mean	*p*_for trend_
Age			<0.001		<0.001		<0.001
18~33 years	1234(33.2)	82.29(66.34)		70.85(46.24)		153.14(108.24)	
34~56 years	1217(32.7)	86.23(68.49)		68.52(44.46)		154.76(107.02)	
≥ 57 years	1266(34.1)	62.45(44.33)		53.70(33.19)		116.15(73.11)	
Sex			0.157		<0.001		0.374
Male	1831(49.3)	85.36(69.79)		65.36(46.11)		150.72(111.54)	
Female	1886(50.7)	68.54(50.71)		63.17(38.24)		131.70(83.76)	
Race			0.003		0.071		0.345
Mexican American	817(22.0)	65.86(43.11)		62.45(35.24)		128.30(74.36)	
Other Hispanic	114(3.1)	68.02(59.93)		59.95(43.64)		127.96(97.15)	
Non-Hispanic White	1835(49.4)	79.54(64.32)		66.62(44.97)		146.16(104.44)	
Non-Hispanic Black	790(21.3)	83.52(68.51)		61.50(41.74)		145.02(105.71)	
Others	161(4.3)	74.87(63.88)		62.91(44.54)		137.78(103.49)	
Current smoking			<0.001		<0.001		<0.001
No	2779(74.8)	55.56(34.14)		52.86(30.27)		108.42(60.85)	
Yes	938(25.2)	139.81(78.72)		97.99(53.45)		237.81(123.55)	
Current drinking			0.241		0.491		0.703
No	1492(40.1)	69.09(53.23)		62.22(39.05)		131.31(88.35)	
Yes	2225(59.9)	82.01(65.87)		65.61(44.31)		147.62(104.86)	
Education level			<0.001		<0.001		<0.001
<High school	923(24.8)	80.03(67.07)		65.76(44.57)		145.78(106.67)	
High school	1139(30.6)	85.56(67.74)		71.12(47.38)		156.68(109.47)	
Social college	1007(27.1)	75.63(58.45)		62.86(39.77)		138.49(93.37)	
College or above	648(17.4)	58.75(37.42)		52.18(28.39)		110.93(62.59)	
Income			0.848		0.621		0.730
Low	817(22.0)	84.69(72.66)		69.30(50.47)		153.99(117.36)	
Middle	1984(53.4)	77.32(61.44)		64.21(41.73)		141.53(98.71)	
High	916(24.6)	68.73(48.11)		59.82(34.37)		128.55(77.44)	
BMI			<0.001		0.258		<0.001
<24.94	1236(33.3)	91.00(75.72)		67.91(46.73)		158.92(117.30)	
24.94~29.92	1241(33.4)	71.50(54.27)		62.39(40.62)		133.89(89.98)	
≥29.93	1240(33.4)	68.02(48.46)		62.46(38.98)		130.47(83.80)	
Regular exercise			0.756		<0.001		0.028
Yes	1431(38.5)	74.83(57.63)		66.44(42.97)		140.95(100.60)	
No	2286(61.5)	78.07(63.68)		62.88(41.83)		141.27(96.09)	
Energy(kcal)			<0.001		<0.001		<0.001
<1662.0	1237(33.3)	71.92(56.77)		61.65(39.32)		133.57(90.55)	
1662.0~2346.5	1240(33.4)	70.54(52.57)		60.30(37.25)		130.84(85.41)	
≥2346.5	1240(33.4)	88.00(71.77)		70.78(48.71)		158.78(115.64)	
Cotinine(ng/ml)			<0.001		<0.001		<0.001
<0.031	1237(33.3)	50.12(19.84)		50.53(24.36)		100.65(41.49)	
0.031~0.738	1240(33.4)	53.51(28.41)		50.48(26.07)		104.00(50.82)	
≥0.738	1240(33.4)	126.77(79.79)		91.70(54.45)		218.47(126.57)	
Diabetes			0.135		0.729		0.289
Yes	445(12.0)	64.02(41.95)		57.69(34.49)		121.71(72.13)	
No	3272(88.0)	78.56(63.43)		65.14(43.19)		143.70(101.70)	
Hypertension			0.848		0.065		0.329
Yes	1360(36.6)	69.75(55.47)		57.96(37.14)		127.71(87.77)	
No	2357(63.4)	80.90(64.28)		67.88(44.62)		148.78(103.99)	
Dyslipidemia			0.899		0.110		0.503
Yes	1492(40.1)	72.49(56.14)		62.76(40.44)		135.25(91.63)	
No	2225(59.9)	79.73(64.59)		65.25(43.49)		144.97(103.29)	
INFLA-score			0.010		<0.001		<0.001
-16~-6	866(23.3)	75.06(59.63)		58.45(38.79)		133.51(94.11)	
-5~5	2121(57.1)	75.58(60.64)		64.12(42.99)		139.70(98.79)	
5~16	730(19.6)	82.51(65.43)		71.50(43.26)		154.01(103.46)	

The levels of hemoglobin biomarkers of AA were presented as mean (standard deviation). BMI, body mass index; Diabetes was defined by a self­reported diagnosis, a hemoglobin A1c level ≥6.5%, a fasting plasma glucose level ≥7.0mmol/L or receiving medications for diabetes; Hypertension was defined by a self-reported diagnosis, the systolic blood pressure ≥140mm Hg, the diastolic blood pressure ≥90mm Hg or receiving medications for hypertension; Dyslipidemia was defined by serum triglyceride ≥2.26 mmol/L, serum cholesterol ≥6.22 mmol/L, low­density lipoprotein cholesterol ≥4.14 mmol/L or receiving medications for dyslipidemia; INFLA-score, low-grade inflammation score.

### AA exposure and cancer mortality

**Figure 1:**
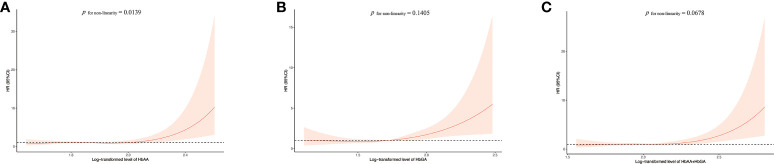
Association between HbAA **(A)**, HbGA **(B)** and HbAA+HbGA **(C)** with cancer mortality in total population. HRs were adjusted for age, sex (male/female), race (Mexican American, other Hispanic, non-Hispanic white, non-Hispanic black or other races), regular exercise (yes/no), body mass index (BMI), education (< 9th Grade, 9-11th Grade, High School Grade, GED or Equivalent, Some College or AA degree, College Graduate or above), income (< $20,000, $20,000-$45,000, $45,000-$75,000, $75,000-$100,000 or > $100,000), current smoker (yes/no), current drinker (yes/no), energy (kcal/d), cotinine (ng/ml), diabetes (yes/no), hypertension (yes/no), dyslipidemia (yes/no) and INFLA-score. HR, hazard ratio.

After a mean of 10.32 years of follow-up from NHANES interview date, a total of 118 participants were reported to die due to cancer. Analysis of CPH regression for the relationship between hemoglobin biomarkers of AA and cancer mortality was presented in **Table 2**. With the potential covariates adjusted, HbAA, HbGA and HbAA + HbGA were found to be positively associated with cancer mortality. In contrast to the lowest quintile of HbAA, the HRs for cancer mortality in the highest quintile were 2.75 (95%CI:1.61-4.70) for model 1, 2.08 (95%CI:1.05-4.14) for model 2, 2.16 (95%CI:1.09-4.30) for model 3 and 2.07 (95%CI:1.04-4.14) for fully adjusted model 4. Moreover, a significant linear correlation was observed between HbGA and cancer mortality (*P*_for trend_ < 0.05), with the HRs in the highest quintile of 2.75 (95%CI:1.62-4.67) for model 1, 2.34 (95%CI:1.27-4.31) for model 2, 2.47 (95%CI:1.34-4.56) for model 3 and 2.39 (95%CI:1.29-4.43) for model 4, compared with the lowest quintile. And the HRs in the highest quintile of HbAA + HbGA were 3.03 (95%CI:1.77-5.21), 2.43 (95%CI:1.26-4.69), 2.58 (95%CI:1.34-4.98) and 2.48 (95%CI:1.28-4.80) for the four models, respectively. Whereas no significant association was found between the hemoglobin biomarkers of AA and all-cause mortality (**Supplementary Table 1**). Further, results for RCS models were showed in **Figure 1**. With the increase of AA biomarkers, the HRs for cancer mortality increased rapidly, presenting a dose-response relationship. Besides, there was a considerable non-linearity association between HbAA and cancer mortality (*P*_for non-linearity_ = 0.0139).

**Table 2:** Multivariate adjusted HRs of HbAA, HbGA and HbAA+HbGA for cancer mortality in total population.

AA Hemoglobin Biomarkers	Case/N	Model 1 ^a^	Model 2 ^b^	Model 3 ^c^	Model 4 ^d^
		HR (95%CI)	HR (95%CI)	HR (95%CI)	HR (95%CI)
**HbAA(pmoL/g Hb)**					
Q1(≤38.9)	32/739	1	1	1	1
Q2(39.0-49.1)	23/741	0.98(0.57-1.67)	0.99(0.58-1.71)	1.01(0.59-1.73)	1.01(0.59-1.74)
Q3(49.2-60.9)	18/749	0.80(0.45-1.42)	0.86(0.48-1.54)	0.87(0.48-1.56)	0.90(0.50-1.62)
Q4(61.0-100.0)	15/742	0.81(0.44-1.51)	0.80(0.43-1.50)	0.82(0.44-1.54)	0.82(0.43-1.53)
Q5(≥101.0)	30/746	2.75(1.61-4.70)	2.08(1.05-4.14)	2.16(1.09-4.30)	2.07(1.04-4.14)
*p*_for trend_		0.014	0.356	0.297	0.341
**HbGA(pmoL/g Hb)**					
Q1(≤34.6)	32/742	1	1	1	1
Q2(34.7-45.9)	16/742	0.70(0.39-1.29)	0.73(0.40-1.35)	0.76(0.41-1.40)	0.75(0.41-1.39)
Q3(46.0-59.9)	24/746	1.23(0.72-2.10)	1.34(0.78-2.31)	1.34(0.78-2.32)	1.35(0.79-2.34)
Q4(60.0-84.7)	17/743	1.14(0.62-2.08)	1.03(0.56-1.91)	1.04(0.56-1.93)	1.04(0.56-1.92)
Q5(≥84.8)	29/744	2.75(1.62-4.67)	2.34(1.27-4.31)	2.47(1.34-4.56)	2.39(1.29-4.43)
*p*_for trend_		<0.001	0.016	0.011	0.014
**HbAA+HbGA**					
Q1(≤75.8)	31/743	1	1	1	1
Q2(75.9-95.9)	21/739	0.99(0.57-1.73)	1.01(0.57-1.76)	1.04(0.59-1.83)	1.05(0.60-1.85)
Q3(96.1-121.4)	18/748	0.91(0.51-1.64)	0.95(0.53-1.72)	0.97(0.54-1.76)	0.99(0.55-1.79)
Q4(121.5-184.5)	19/743	1.27(0.71-2.26)	1.19(0.66-2.15)	1.22(0.67-2.21)	1.23(0.68-2.23)
Q5(≤184.7)	29/744	3.03(1.77-5.21)	2.43(1.26-4.69)	2.58(1.34-4.98)	2.48(1.28-4.80)
*p*_for trend_		0.001	0.044	0.031	0.039

The levels of hemoglobin biomarkers of AA were presented as HRs (95% CIs). **^a^** Model 1 adjusted for age, sex, and race. **^b^** Model 2 additionally adjusted for smoking and drinking status, education level, income, BMI, regular exercise, energy and cotinine. **^c^** Model 3 further adjusted disease status of diabetes, hypertension and dyslipidemia. **^d^** Model 4 finally adjusted for INFLA-score. In addition, log-transformed levels of HbAA, HbGA and HbAA+HbGA were adjusted when analyzing the association between hemoglobin biomarkers of AA and cancer mortality. Q, quintile; HR, hazard ratio; CI, confidence interval.

### Mediated effects of INFLA-score

**Figure 2:**
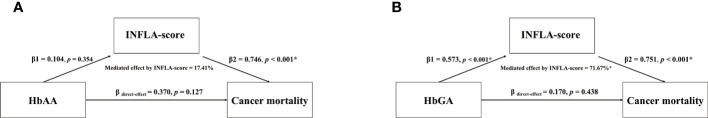
Mediated effects of INFLA-score on the association between HbAA **(A)** and HbGA **(B)** and cancer mortality. Mediation analysis was adjusted for age, sex (male/female), race (Mexican American, other Hispanic, non-Hispanic white, non-Hispanic black or other races), regular exercise (yes/no), body mass index (BMI), education (< 9th Grade, 9-11th Grade, High School Grade, GED or Equivalent, Some College or AA degree, College Graduate or above), income (< $20,000, $20,000-$45,000, $45,000-$75,000, $75,000-$100,000 or > $100,000), current smoker (yes/no), current drinker (yes/no), energy (kcal/d), cotinine (ng/ml), diabetes (yes/no), hypertension (yes/no), dyslipidemia (yes/no) and INFLA-score. β1, the indirect effect of AA biomarkers on INFLA-score; β2, the indirect effect of INFLA-score on cancer mortality; β _direct-effect_, the direct effect of AA biomarkers on cancer mortality; *p < 0.05.

Mediated effects of INFLA-score on the relationship of HbAA and HbGA with cancer mortality were shown in **Figure 2**. The β1 and β2 values represented the indirect effect of AA biomarkers on INFLA-score and INFLA-score on cancer mortality, respectively. Moreover, the β _direct-effect_ value represented the direct effect of AA biomarkers on cancer mortality. The results showed that INFLA-score significantly mediated 71.67% for HbGA induced increased cancer mortality risk, and the direct effect of HbGA on cancer mortality showed no significance (*P* = 0.438), suggesting a complete mediated role of INFLA-score on the association between HbGA and cancer mortality. However, mediated effect of INFLA-score for HbAA induced increased cancer mortality risk showed no significance (*P* > 0.05).

### Sensitivity analysis

In the first sensitivity analysis, after participants with less than two years of follow-up were excluded, the association between AA biomarkers and cancer mortality was still significant, suggesting that the results were relatively robust and were not affected by severe disease (**Supplementary Table 2**). In the second sensitivity analysis, we performed our analysis in the non-cancer group using step-by-step method to identify whether the results were stable after excluding the effect of cancer disease. The results showed a robust association between AA biomarkers and cancer mortality, which was consistent with our results of the total sample (**Supplementary Table 3**). In the third sensitivity analysis, the results suggested that behavior of smoking or status of diseases (hypertension, dyslipidemia and diabetes) could not interact with the effect of AA exposure on cancer mortality (**Supplementary Table 4**).

## Discussion

In the large cohort study, we innovatively found that hemoglobin biomarkers of AA were positively associated with cancer mortality among the U.S. general population after adjusting sociodemographic factors, lifestyle factors and disease indicators. Interestingly, inflammation played a mediated role in this process. Moreover, consistent results could also be observed in subgroup sensitivity analyses, and behavior of smoke or status of diseases (hypertension, dyslipidemia and diabetes) could not modify the association, indicating that the results were considerably stable and robust. Therefore, our study provided direct evidence for the effects of AA exposure on cancer mortality in general population and reminded the public to pay more attention to AA exposure.

There have been plenty of studies demonstrating that AA exposure was associated with the development of inflammation. In animal researches, exposure to different doses of AA could significantly increase the levels of inflammatory cytokines in serum or plasma (27–29). Additionally, vitro studies showed that AA treatment could induce expression of pro-inflammatory factors including interleukin-6 (IL-6), interleukin-1β (IL-1β), tumor necrosis factor-α (TNF-α) and granulocyte colony-stimulating factor (G-CSF) *via* nuclear factor-κB (NF-κB) pathway (30, 31). Whereas only a few population-based studies on AA induced inflammation were documented. A pilot study showed that intakes of food containing AA could elevate levels of CRP in plasma, and a recent epidemiological study mentioned that AA exposure in daily life was positively associated with systemic inflammation in general population (24), which was consistent with our study.

Inflammatory markers in blood circulation are risk factors of numerous diseases, including atherosclerosis, neurological conditions and neoplastic progression (32–34). IL-1β was reported to be associated with gastric cancer (35), and TNF-α could function as a tumor promoter in the development of cancer (36–38). Noteworthily, NF-κB played a vitally important role in the progression of inflammation-associated tumors by promoting growth, survival and vascularization of carcinoma cells (39). The Cellular and immune changes caused by inflammatory response can lead to repeated tissue damage and tissue repair (21), which could interact with DNA in proliferating epithelial cells and result in permanent genomic mutations (33), in the environment of abundant reactive oxygen species (ROS) and reactive nitrogen species (RNS) induced by inflammatory cells (40). Indeed, the inflammatory environment contributes to cell proliferation (38, 41), survival (42) and migration (43), all of which provides the base for tumorigenesis (44). Additionally, indirect damage by inflammation such as apoptosis (45) and necrosis (46), can significantly affect homeostasis and generate a fit microenvironment for tumor growth (47). Taken together, AA could induce releases of inflammatory factors, and the latter were associated with the development of tumors. Therefore, for the first time, we took inflammatory indicators into account, to investigate the impact of AA exposure on cancer mortality in general population and found that AA biomarkers were positively associated with cancer mortality, with INFLA-score playing a mediated role in this process. In addition, to explore what types of cancer were more prone to be affected by AA, we performed a logistic regression analysis between AA biomarkers and specific cancer. We found HbGA was positively associated with uterine cancer among women after adjusting potential covariates (**Supplementary Table 5**). Compared to the lowest HbGA group, the OR (95%CI) of the highest group was 4.26 (95%CI: 1.04-17.54) for prevalence of uterine cancer. This result was consistent with a prospective cohort study illustrating that high acrylamide consumers had an increased risk for endometrial cancer (12), and some animal studies demonstrating that exposure to glycidamide could induce uterine adenocarcinoma and endometrial hyperplasia in female rats (48, 49). It has been reported that acrylamide exposure could reduce serum progesterone and estradiol concentrations in female rats (50), and a recent Canada case-control study found a relationship between low estrogens and high endometrial cancer risk (51), which partly supported our finding. Our results are in conflict with some epidemiological studies demonstrating that dietary intakes of AA were not associated with some tissue-specific cancers including lung, liver, breast and gastrointestinal cancer in Japanese population (15–18). A possible explanation for these inverse results is that intakes of AA in these studies were only estimated from food frequency questionnaire (FFQ), which could lead to inaccurate calculation of AA and thus cause insufficient correlation assessment. Nevertheless, A Swedish prospective cohort study found a positive association between AA hemoglobin adducts and risk of breast cancer with an estimated incidence rate ratio of 2.7 (95%CI:1.1-6.6) (13), which was in line with our findings.

Furthermore, in addition to inflammatory factors, other potential mechanisms may also explain the process of cancer induced by AA. Human and animal studies have illustrated that exposure to AA could cause DNA damage, activate Kirsten-ras (KRAS) mutation, promote cell proliferation and thus lead to carcinogenesis (52–54). Besides, there were also studies demonstrating that carcinogenicity of AA depended on metabolic conversion to GA (55, 56). GA-induced adducts might be involved in spontaneous depurination or tissue-specific repair, which could induce tumors (57). Further, a prospective pilot study of 62573 women illustrated that ovarian cancer might be caused through effects of AA on single nucleotide polymorphisms (SNPs) of genes involved in sex hormones system (*P*_for interaction_ = 0.04) (58). The studies above, through different mechanisms, supported our finding that AA was positively associated with cancer mortality in general population.

To our best knowledge, this study was the first one to confirm the significant association between AA exposure and cancer mortality in general population, and proposed that inflammation played a mediated role in this process. We took advantage of the reliable blood indicators of AA and avoided the miscalculation of dietary AA intakes from FFQ. Our study, therefore, could highlight public concern about the effects of widespread exposure to AA on residents’ health, and provided an alternative method to reduce cancer mortality risk induced by AA through anti-inflammatory effects. The government should formulate relevant policies or recommendations to authorize the restriction or control of public exposure to AA from various sources, in particular, smoking or intakes of AA-rich foods such as fried foods and coffee. The association between hemoglobin biomarkers of AA and cancer mortality in our study was relatively stable and robust after adjusting a series of covariates. However, there were still several limitations that should be taken into consideration when interpreting these findings. Firstly, though we took advantage of this representative cohort and comprehensively evaluated the effects of AA exposure on cancer mortality, the survival of cancer depends on many factors, such as type, stage and site of cancer, as well as age and sex of patients. Therefore, future studies are needed to elucidate the possible mechanism in the association between AA exposure and specific cancer. Secondly, the concentration of AA biomarkers was only tested once, which may cause insufficient correlation assessment. Thirdly, since we adjusted a large number of covariables to reduce residual confounders, the risk of over-adjustment may be increased. Finally, this cohort was mainly representative of the U.S. general population, so the results should be verified in other populations.

## Conclusion

In conclusion, exposure of AA is positively associated with cancer mortality in adult American population, and INFLA-score plays a mediated role in the association. It can emphasize public concern about AA exposure and remind people to stay away from tobacco smoke and AA-rich foods, and have a healthy diet.

## Data availability statement

Publicly available datasets were analyzed in this study. This data can be found here: https://wwwn.cdc.gov/nchs/nhanes/Default.aspx.

## Author contributions

WG: Methodology, Formal analysis, Writing-Original Draft. JZ: Methodology, Formal analysis. CR and YG: Methodology, Investigation. TZ, YL and WW: Investigation, Validation. SH and CS: Validation. CW, WJ and JZ: Conceptualization, Writing-Original Draft, Writing-Review & Editing. All authors contributed to the article and approved the submitted version.

## Funding

This work was supported by HMU Marshal Initiative Funding [HMUMIF-21011 to Wenbo Jiang], China Postdoctoral Natural Science Foundation [2021M701021 to Wenbo Jiang] and National Natural Science Foundation of China [82000081 to Junfei Zhao, 81872615 to Shaoying Hou].

## Acknowledgments

We appreciate all the participants and staff of NHANES 2003-2014 for their precious contributions.

## Conflict of interest

The authors declare that the research was conducted in the absence of any commercial or financial relationships that could be construed as a potential conflict of interest.

## Publisher’s note

All claims expressed in this article are solely those of the authors and do not necessarily represent those of their affiliated organizations, or those of the publisher, the editors and the reviewers. Any product that may be evaluated in this article, or claim that may be made by its manufacturer, is not guaranteed or endorsed by the publisher.

## References

[1] Friedman M. Chemistry, biochemistry, and safety of acrylamide. A review J Agric Food Chem (2003) 51(16):4504–26. doi: 10.1021/jf030204+ 14705871

[2] Lin CY Lee HL Chen YC Lien GW Lin LY Wen LL. Positive association between urinary levels of 8-hydroxydeoxyguanosine and the acrylamide metabolite n-acetyl-S-(propionamide)-cysteine in adolescents and young adults. J Hazard Mater (2013) 261:372–7. doi: 10.1016/j.jhazmat.2013.06.069 23959257

[3] Sharp D. Acrylamide in food. Lancet (2003) 361(9355):361–2. doi: 10.1016/s0140-6736(03)12442-7 12573369

[4] Koszucka A Nowak A Nowak I Motyl I. Acrylamide in human diet, its metabolism, toxicity, inactivation and the associated European union legal regulations in food industry. Crit Rev Food Sci Nutr (2020) 60(10):1677–92. doi: 10.1080/10408398.2019.1588222 30907623

[5] Calleman CJ Bergmark E Costa LG. Acrylamide is metabolized to glycidamide in the rat: evidence from hemoglobin adduct formation. Chem Res Toxicol (1990) 3(5):406–12. doi: 10.1021/tx00017a004 2133091

[6] Fennell TR Sumner SC Snyder RW Burgess J Spicer R Bridson WE. Metabolism and hemoglobin adduct formation of acrylamide in humans. Toxicol Sci (2005) 85(1):447–59. doi: 10.1093/toxsci/kfi069 15625188

[7] Zhang Y Huang M Zhuang P Jiao J Chen X Wang J. Exposure to acrylamide and the risk of cardiovascular diseases in the national health and nutrition examination survey 2003-2006. Environ Int (2018) 117:154–63. doi: 10.1016/j.envint.2018.04.047 29753146

[8] Wang B Cheng M Yang S Qiu W Li W Zhou Y. Exposure to acrylamide and reduced heart rate variability: The mediating role of transforming growth factor-β. J Hazard Mater (2020) 395:122677. doi: 10.1016/j.jhazmat.2020.122677 32339852

[9] Hogervorst JG Baars BJ Schouten LJ Konings EJ Goldbohm RA van den Brandt PA. The carcinogenicity of dietary acrylamide intake: A comparative discussion of epidemiological and experimental animal research. Crit Rev Toxicol (2010) 40(6):485–512. doi: 10.3109/10408440903524254 20170357

[10] Imai T Cho YM Hasumura M Hirose M. Enhancement by acrylamide of n-methyl-N-nitrosourea-induced rat mammary tumor development-possible application for a model to detect co-modifiers of carcinogenesis. Cancer Lett (2005) 230(1):25–32. doi: 10.1016/j.canlet.2004.12.019 16253758

[11] Zhang B Shao H Wang XH Chen X Li ZS Cao P. Acrylamide-induced subacute neurotoxic effects on the cerebral cortex and cerebellum at the synapse level in rats. BioMed Environ Sci (2017) 30(6):432–43. doi: 10.3967/bes2017.057 28705267

[12] Wilson KM Mucci LA Rosner BA Willett WC. A prospective study on dietary acrylamide intake and the risk for breast, endometrial, and ovarian cancers. Cancer Epidemiol Biomarkers Prev (2010) 19(10):2503–15. doi: 10.1158/1055-9965.epi-10-0391 PMC2952046 20693310

[13] Olesen PT Olsen A Frandsen H Frederiksen K Overvad K Tjønneland A. Acrylamide exposure and incidence of breast cancer among postmenopausal women in the Danish diet, cancer and health study. Int J Cancer (2008) 122(9):2094–100. doi: 10.1002/ijc.23359 18183576

[14] Hogervorst JG Schouten LJ Konings EJ Goldbohm RA van den Brandt PA. A prospective study of dietary acrylamide intake and the risk of endometrial, ovarian, and breast cancer. Cancer Epidemiol Biomarkers Prev (2007) 16(11):2304–13. doi: 10.1158/1055-9965.epi-07-0581 18006919

[15] Kotemori A Ishihara J Zha L Liu R Sawada N Iwasaki M. Dietary acrylamide intake and risk of breast cancer: The Japan public health center-based prospective study. Cancer Sci (2018) 109(3):843–53. doi: 10.1111/cas.13496 PMC5834785 29288560

[16] Liu R Sobue T Kitamura T Kitamura Y Ishihara J Kotemori A. Dietary acrylamide intake and risk of esophageal, gastric, and colorectal cancer: The Japan public health center-based prospective study. Cancer Epidemiol Biomarkers Prev (2019) 28(9):1461–8. doi: 10.1158/1055-9965.epi-18-1259 31186264

[17] Liu R Zha L Sobue T Kitamura T Ishihara J Kotemori A. Dietary acrylamide intake and risk of lung cancer: The Japan public health center based prospective study. Nutrients (2020) 12(8):2417. doi: 10.3390/nu12082417 PMC7468968 32806637

[18] Zha L Sobue T Kitamura T Kitamura Y Ishihara J Kotemori A. Dietary acrylamide intake and the risk of liver cancer: The Japan public health center-based prospective study. Nutrients (2020) 12(9):2503. doi: 10.3390/nu12092503 PMC7551605 32825036

[19] Greten FR Grivennikov SI. Inflammation and cancer: Triggers, mechanisms, and consequences. Immunity (2019) 51(1):27–41. doi: 10.1016/j.immuni.2019.06.025 31315034 PMC6831096

[20] Singh R Mishra MK Aggarwal H. Inflammation, immunity, and cancer. Mediators Inflammation (2017) 2017:6027305. doi: 10.1155/2017/6027305 PMC5695028 29234189

[21] Singh N Baby D Rajguru JP Patil PB Thakkannavar SS Pujari VB. Inflammation and cancer. Ann Afr Med (2019) 18(3):121–6. doi: 10.4103/aam.aam_56_18 PMC6704802 31417011

[22] Bonaccio M Di Castelnuovo A Pounis G De Curtis A Costanzo S Persichillo M. A score of low-grade inflammation and risk of mortality: prospective findings from the moli-sani study. Haematologica (2016) 101(11):1434–41. doi: 10.3324/haematol.2016.144055 PMC5394885 27742767

[23] Izzi B Gianfagna F Yang WY Cludts K De Curtis A Verhamme P. Variation of PEAR1 DNA methylation influences platelet and leukocyte function. Clin Epigenet (2019) 11(1):151. doi: 10.1186/s13148-019-0744-8 PMC6820903 31665082

[24] Wang B Wang X Yang S Cheng M Zhou Y Zhou M. Acrylamide exposure and pulmonary function reduction in general population: The mediating effect of systemic inflammation. Sci Total Environ (2021) 778:146304. doi: 10.1016/j.scitotenv.2021.146304 34030393

[25] Shan Z Rehm CD Rogers G Ruan M Wang DD Hu FB. Trends in dietary carbohydrate, protein, and fat intake and diet quality among US adults, 1999-2016. JAMA (2019) 322(12):1178–87. doi: 10.1001/jama.2019.13771 PMC6763999 31550032

[26] Vesper HW Caudill SP Osterloh JD Meyers T Scott D Myers GL. Exposure of the U.S. population to acrylamide in the national health and nutrition examination survey 2003-2004. Environ Health Perspect (2010) 118(2):278–83. doi: 10.1289/ehp.0901021 PMC2831930 20123601

[27] Abdel-Daim MM Abd Eldaim MA Hassan AG. Trigonella foenum-graecum ameliorates acrylamide-induced toxicity in rats: Roles of oxidative stress, proinflammatory cytokines, and DNA damage. Biochem Cell Biol (2015) 93(3):192–8. doi: 10.1139/bcb-2014-0122 25607344

[28] Alturfan AA Tozan-Beceren A Sehirli AO Demiralp E Sener G Omurtag GZ. Resveratrol ameliorates oxidative DNA damage and protects against acrylamide- induced oxidative stress in rats. Mol Biol Rep (2012) 39(4):4589–96. doi: 10.1007/s11033-011-1249-5 21947844

[29] Zhang L Wang E Chen F Yan H Yuan Y. Potential protective effects of oral administration of allicin on acrylamide-induced toxicity in male mice. Food Funct (2013) 4(8):1229–36. doi: 10.1039/c3fo60057b 23760623

[30] Pan X Wu X Yan D Peng C Rao C Yan H. Acrylamide-induced oxidative stress and inflammatory response are alleviated by n-acetylcysteine in PC12 cells: Involvement of the crosstalk between Nrf2 and NF-κB pathways regulated by MAPKs. Toxicol Lett (2018) 288:55–64. doi: 10.1016/j.toxlet.2018.02.002 29426002

[31] Zhao M Lewis Wang FS Hu X Chen F Chan HM. Acrylamide-induced neurotoxicity in primary astrocytes and microglia: Roles of the Nrf2-ARE and NF-κB pathways. Food Chem Toxicol (2017) 106:25–35 doi: 10.1016/j.fct.2017.05.007 28526328

[32] Lind L. Circulating markers of inflammation and atherosclerosis. Atherosclerosis (2003) 169(2):203–14. doi: 10.1016/s0021-9150(03)00012-1 12921971

[33] Coussens LM Werb Z. Inflammation and cancer. Nature (2002) 420(6917):860–7. doi: 10.1038/nature01322 PMC2803035 12490959

[34] Samuels MA. Inflammation and neurological disease. Curr Opin Neurol (2004) 17(3):307–9. doi: 10.1097/00019052-200406000-00012 15167066

[35] El-Omar EM Carrington M Chow WH McColl KE Bream JH Young HA. Interleukin-1 polymorphisms associated with increased risk of gastric cancer. Nature (2000) 404(6776):398–402. doi: 10.1038/35006081 10746728

[36] Balkwill F. Tumor necrosis factor or tumor promoting factor? Cytokine Growth Factor Rev (2002) 13(2):135–41. doi: 10.1016/S1359-6101(01)00020-X 11900989

[37] Arnott CH Scott KA Moore RJ Hewer A Phillips DH Parker P. Tumour necrosis factor-alpha mediates tumour promotion *via* a PKC alpha- and AP-1-dependent pathway. Oncogene (2002) 21(31):4728–38. doi: 10.1038/sj.onc.1205588 12101411

[38] Pikarsky E Porat RM Stein I Abramovitch R Amit S Kasem S. NF-kappaB functions as a tumour promoter in inflammation-associated cancer. Nature (2004) 431(7007):461–6. doi: 10.1038/nature02924 15329734

[39] Karin M. Nuclear factor-kappaB in cancer development and progression. Nature (2006) 441(7092):431–6. doi: 10.1038/nature04870 16724054

[40] Maeda H Akaike T. Nitric oxide and oxygen radicals in infection, inflammation, and cancer. Biochemistry(Mosc) (1998) 63(7):854–65. 9721338

[41] Kiraly O Gong G Olipitz W Muthupalani S Engelward BP. Inflammation induced cell proliferation potentiates DNA damage-induced mutations in vivo. PloS Genet (2015) 11(2):e1004901. doi: 10.1371/journal.pgen.1004901 25647331 PMC4372043

[42] Burns JM Summers BC Wang Y Melikian A Berahovich R Miao Z. A novel chemokine receptor for SDF-1 and I TAC involved in cell survival, cell adhesion, and tumor development. J Exp Med (2006) 203(9):2201–13. doi: 10.1084/jem.20052144 PMC2118398 16940167

[43] Wedmore CV Williams TJ. Control of vascular permeability by polymorphonuclear leukocytes in inflammation. Nature (1981) 289(5799):646–50. doi: 10.1038/289646a0 7464931

[44] Allavena P Garlanda C Borrello MG Sica A Mantovani A. Pathways connecting inflammation and cancer. Curr Opin Genet Dev (2008) 18(1):3–10. doi: 10.1016/j.gde.2008.01.003 18325755

[45] Savill JS Wyllie AH Henson JE Walport MJ Henson PM Haslett C. Macrophage phagocytosis of aging neutrophils in inflammation. programmed cell death in the neutrophil leads to its recognition by macrophages. J Clin Invest (1989) 83(3):865–75. doi: 10.1172/JCI113970 PMC303760 2921324

[46] Sosna J Voigt S Mathieu S Lange A Thon L Davarnia P. TNF-induced necroptosis and PARP-1-mediated necrosis represent distinct routes to programmed necrotic cell death. Cell Mol Life Sci (2014) 71(2):331–48. doi: 10.1007/s00018-013-1381-6 PMC3889832 23760205

[47] Kay J Thadhani E Samson L Engelward B. Inflammation-induced DNA damage, mutations and cancer. DNA Repair(Amst) (2019) 83:102673. doi: 10.1016/j.dnarep.2019.102673 31387777 PMC6801086

[48] Johnson KA Gorzinski SJ Bodner KM Campbell RA Wolf CH Friedman MA. Chronic toxicity and oncogenicity study on acrylamide incorporated in the drinking water of Fischer 344 rats. Toxicol Appl Pharmacol (1986) 85(2):154–68. doi: 10.1016/0041-008x(86)90109-2 3764902

[49] Beland FA Olson GR Mendoza MCB Marques MM Doerge DR. Carcinogenicity of glycidamide in B6C3F1 mice and F344/N rats from a two-year drinking water exposure. Food Chem Toxicol (2015) 86:104–15. doi: 10.1016/j.fct.2015.09.017 PMC5066397 26429628

[50] Aldawood N Alrezaki A Alanazi S Amor N Alwasel S Sirotkin A. Acrylamide impairs ovarian function by promoting apoptosis and affecting reproductive hormone release, steroidogenesis and autophagy-related genes: An *in vivo* study. Ecotoxicol Environ Saf (2020) 197:110595. doi: 10.1016/j.ecoenv.2020.110595 32304918

[51] Friedenreich CM Derksen JWG Speidel T Brenner DR Heer E Courneya KS. Case-control study of endogenous sex steroid hormones and risk of endometrial cancer. Cancer Causes Control (2020) 31(2):161–71. doi: 10.1007/s10552-019-01260-5 31865473

[52] Wang B Qiu W Yang S Cao L Zhu C Ma J. Acrylamide exposure and oxidative DNA damage, lipid peroxidation, and fasting plasma glucose alteration: Association and mediation analyses in Chinese urban adults. Diabetes Care (2020) 43(7):1479–86. doi: 10.2337/dc19-2603 32345652

[53] Lafferty JS Kamendulis LM Kaster J Jiang J Klaunig JE. Subchronic acrylamide treatment induces a tissue-specific increase in DNA synthesis in the rat. Toxicol Lett (2004) 154(1-2):95–103. doi: 10.1016/j.toxlet.2004.07.008 15475183

[54] Hogervorst JG de Bruijn-Geraets D Schouten LJ van Engeland M de Kok TM Goldbohm RA. Dietary acrylamide intake and the risk of colorectal cancer with specific mutations in KRAS and APC. Carcinogenesis (2014) 35(5):1032–8. doi: 10.1093/carcin/bgu002 24398672

[55] Beland FA Mellick PW Olson GR Mendoza MC Marques MM Doerge DR. Carcinogenicity of acrylamide in B6C3F(1) mice and F344/N rats from a 2-year drinking water exposure. Food Chem Toxicol (2013) 51:149–59. doi: 10.1016/j.fct.2012.09.017 23009883

[56] Von Tungeln LS Doerge DR Gamboa da Costa G Matilde Marques M Witt WM Koturbash I. Tumorigenicity of acrylamide and its metabolite glycidamide in the neonatal mouse bioassay. Int J Cancer (2012) 131(9):2008–15. doi: 10.1002/ijc.27493 PMC4810677 22336951

[57] Manjanatha MG Guo LW Shelton SD Doerge DR. Acrylamide-induced carcinogenicity in mouse lung involves mutagenicity: cII gene mutations in the lung of big blue mice exposed to acrylamide and glycidamide for up to 4 weeks. Environ Mol Mutagen (2015) 56(5):446–56. doi: 10.1002/em.21939 25639614

[58] Hogervorst JGF van den Brandt PA Godschalk RWL van Schooten FJ Schouten LJ. Interactions between dietary acrylamide intake and genes for ovarian cancer risk. Eur J Epidemiol (2017) 32(5):431–41. doi: 10.1007/s10654-017-0244-0 PMC5506210 28391539

