# Supplementary material for: The association between biomarkers of acrylamide and cancer mortality in U.S. adult population: Evidence from NHANES 2003-2014
Source: Front Oncol. 2022 Sep 28;12:970021. doi: 10.3389/fonc.2022.970021 (PMC9554530; doi:10.3389/fonc.2022.970021)
Supplement: Supplementary file 1 [file DataSheet_1.docx]

**Supplementary table 1**

Multivariate adjusted HRs of HbAA, HbGA and HbAA+HbGA for all-cause mortality in total population.

| **AA Hemoglobin Biomarkers** | **Case/N** | **Model 1 ^a^** | **Model 2 ^b^** | **Model 3 ^c^** | **Model 4 ^d^** |
| --- | --- | --- | --- | --- | --- |
|  |  | **HR (95%CI)** | **HR (95%CI)** | **HR (95%CI)** | **HR (95%CI)** |
| **HbAA(pmoL/g Hb)** | |  |  |  |  |
| Q1(<38.9) | 147/739 | 1 | 1 | 1 | 1 |
| Q2(39.0-49.1) | 101/741 | 0.90(0.70-1.16) | 0.93(0.72-1.20) | 0.97(0.75-1.25) | 0.97(0.75-1.25) |
| Q3(49.2-60.9) | 88/749 | 0.81(0.62-1.05) | 0.85(0.65-1.11) | 0.88(0.67-1.15) | 0.91(0.69-1.19) |
| Q4(61.0-100.0) | 88/742 | 0.97(0.74-1.27) | 0.96(0.73-1.26) | 1.01(0.77-1.32) | 0.99(0.75-1.30) |
| Q5(>101.0) | 89/746 | 1.74(1.32-2.30) | 1.36(0.95-1.94) | 1.40(0.98-2.01) | 1.32(0.92-1.90) |
| *P* _for trend_ |  | 0.009 | 0.523 | 0.359 | 0.468 |
| **HbGA(pmoL/g Hb)** | |  |  |  |  |
| Q1(<34.6) | 152/742 | 1 | 1 | 1 | 1 |
| Q2(34.7-45.9) | 105/742 | 0.88(0.69-1.13) | 0.91(0.71-1.17) | 0.93(0.73-1.20) | 0.92(0.72-1.19) |
| Q3(46.0-59.9) | 100/746 | 0.95(0.74-1.23) | 1.00(0.77-1.30) | 1.01(0.78-1.31) | 1.01(0.78-1.31) |
| Q4(60.0-84.7) | 76/743 | 0.91(0.69-1.21) | 0.84(0.63-1.12) | 0.85(0.63-1.13) | 0.84(0.63-1.12) |
| Q5(>84.8) | 80/744 | 1.42(1.07-1.88) | 1.12(0.81-1.55) | 1.14(0.82-1.57) | 1.08(0.78-1.50) |
| *P* _for trend_ |  | 0.101 | 0.956 | 0.903 | 0.923 |
| **HbAA+HbGA** | |  |  |  |  |
| Q1(<75.8) | 155/743 | 1 | 1 | 1 | 1 |
| Q2(75.9-95.9) | 102/739 | 0.92(0.71-1.17) | 0.96(0.74-1.23) | 0.99(0.77-1.27) | 0.99(0.77-1.27) |
| Q3(96.1-121.4) | 87/748 | 0.76(0.58-1.00) | 0.78(0.60-1.03) | 0.82(0.62-1.07) | 0.83(0.63-1.08) |
| Q4(121.5-184.5) | 83/743 | 1.00(0.76-1.31) | 0.96(0.73-1.27) | 0.98(0.74-1.30) | 0.99(0.75-1.31) |
| Q5(>184.7) | 86/744 | 1.69(1.28-2.23) | 1.32(0.94-1.86) | 1.36(0.97-1.91) | 1.28(0.91-1.81) |
| *P* _for trend_ |  | 0.015 | 0.641 | 0.498 | 0.625 |

The levels of hemoglobin biomarkers of AA were presented as HRs (95% CIs). **^a^** Model 1 adjusted for age, sex, and race. **^b^** Model 2 additionally adjusted for smoking and drinking status, education level, income, BMI, regular exercise, energy and cotinine. **^c^** Model 3 further adjusted disease status of diabetes, hypertension and dyslipidemia. **^d^** Model 4 finally adjusted for INFLA-score. In addition, log-transformed levels of HbAA, HbGA, and HbAA+HbGA were adjusted when analyzing the association between hemoglobin biomarkers of AA and all-cause mortality. Q, quintile; HR, hazard ratio; CI, confidence interval.

**Supplementary table 2**

Multivariate adjusted HRs of HbAA, HbGA and HbAA+HbGA for cancer mortality in population with follow-up time more than two years.

| **AA Hemoglobin Biomarkers** | **Case/N** | **Model 1 ^a^** | **Model 2 ^b^** | **Model 3 ^c^** | **Model 4 ^d^** |
| --- | --- | --- | --- | --- | --- |
|  |  | **HR (95%CI)** | **HR (95%CI)** | **HR (95%CI)** | **HR (95%CI)** |
| **HbAA(pmoL/g Hb)** | |  |  |  |  |
| Q1(≤38.9) | 26/728 | 1 | 1 | 1 | 1 |
| Q2(39.0-49.2) | 18/738 | 0.93(0.51-1.69) | 0.95(0.52-1.74) | 0.96(0.53-1.77) | 0.97(0.53-1.77) |
| Q3(49.3-60.8) | 16/730 | 0.85(0.45-1.58) | 0.92(0.49-1.74) | 0.94(0.50-1.78) | 0.96(0.51-1.81) |
| Q4(60.9-100.0) | 13/735 | 0.85(0.43-1.66) | 0.84(0.42-1.66) | 0.85(0.43-1.70) | 0.85(0.43-1.69) |
| Q5(≥101.0) | 29/735 | 3.13(1.77-5.53) | 2.33(1.11-4.88) | 2.40(1.14-5.02) | 2.35(1.12-4.95) |
| *P* _for trend_ |  | 0.003 | 0.208 | 0.180 | 0.198 |
| **HbGA(pmoL/g Hb)** | |  |  |  |  |
| Q1(≤34.6) | 25/725 | 1 | 1 | 1 | 1 |
| Q2(34.7-46.0) | 14/741 | 0.76(0.39-1.47) | 0.80(0.41-1.56) | 0.83(0.43-1.62) | 0.83(0.43-1.61) |
| Q3(46.1-59.9) | 20/732 | 1.26(0.70-2.29) | 1.39(0.76-2.54) | 1.39(0.76-2.56) | 1.40(0.76-2.56) |
| Q4(60.0-84.8) | 16/733 | 1.29(0.68-2.45) | 1.14(0.59-2.21) | 1.16(0.60-2.24) | 1.15(0.59-2.23) |
| Q5(≥84.9) | 27/735 | 3.04(1.72-5.40) | 2.46(1.27-4.80) | 2.58(1.32-5.02) | 2.53(1.29-4.95) |
| *P* _for trend_ |  | <0.001 | 0.014 | 0.011 | 0.013 |
| **HbAA+HbGA** | |  |  |  |  |
| Q1(≤75.8) | 25/731 | 1 | 1 | 1 | 1 |
| Q2(75.9-96.1) | 17/736 | 0.97(0.52-1.80) | 0.99(0.53-1.86) | 1.03(0.55-1.94) | 1.04(0.55-1.94) |
| Q3(96.2-121.2) | 16/730 | 0.96(0.51-1.82) | 1.01(0.54-1.92) | 1.04(0.55-1.97) | 1.04(0.55-1.98) |
| Q4(121.3-184.5) | 17/735 | 1.34(0.71-2.50) | 1.23(0.65-2.35) | 1.26(0.66-2.40) | 1.26(0.66-2.41) |
| Q5(≥184.7) | 27/734 | 3.29(1.84-5.89) | 2.47(1.21-5.03) | 2.59(1.27-5.28) | 2.54(1.24-5.21) |
| *P* _for trend_ |  | <0.001 | 0.043 | 0.034 | 0.039 |

The levels of hemoglobin biomarkers of AA were presented as HRs (95% CIs). **^a^** Model 1 adjusted for age, sex, and race. **^b^** Model 2 additionally adjusted for smoking and drinking status, education level, income, BMI, regular exercise, energy and cotinine. **^c^** Model 3 further adjusted disease status of diabetes, hypertension and dyslipidemia. **^d^** Model 4 finally adjusted for INFLA-score. In addition, log-transformed levels of HbAA, HbGA, and HbAA+HbGA were adjusted when analyzing the association between hemoglobin biomarkers of AA and cancer mortality. Q, quintile; HR, hazard ratio; CI, confidence interval.

**Supplement table 3**

Multivariate adjusted HRs of HbAA, HbGA and HbAA+HbGA for cancer mortality in the non-cancer population.

| **AA Hemoglobin Biomarkers** | **Case/N** | **Model 1 ^a^** | **Model 2 ^b^** | **Model 3 ^c^** | **Model 4 ^d^** |
| --- | --- | --- | --- | --- | --- |
|  |  | **HR (95%CI)** | **HR (95%CI)** | **HR (95%CI)** | **HR (95%CI)** |
| **HbAA(pmoL/g Hb)** | |  |  |  |  |
| Q1(≤39.2) | 19/684 | 1 | 1 | 1 | 1 |
| Q2(39.3-49.6) | 13/693 | 0.95(0.47-1.94) | 0.95(0.47-1.94) | 0.97(0.47-1.99) | 0.97(0.48-1.99) |
| Q3(49.7-61.6) | 16/687 | 1.23(0.63-2.41) | 1.24(0.63-2.48) | 1.26(0.63-2.50) | 1.23(0.62-2.45) |
| Q4(61.7-101.0) | 9/684 | 0.93(0.44-1.98) | 0.83(0.38-1.82) | 0.85(0.39-1.86) | 0.85(0.39-1.87) |
| Q5(≥102.0) | 27/695 | 3.64(1.93-6.86) | 3.43(1.53-7.69) | 3.65(1.63-8.17) | 3.74(1.67-8.36) |
| *P* _for trend_ |  | 0.001 | 0.048 | 0.035 | 0.033 |
| **HbGA(pmoL/g Hb)** | |  |  |  |  |
| Q1(≤35.0) | 20/686 | 1 | 1 | 1 | 1 |
| Q2(35.1-46.4) | 11/689 | 0.76(0.36-1.60) | 0.86(0.40-1.82) | 0.90(0.42-1.91) | 0.90(0.42-1.91) |
| Q3(46.5-60.6) | 16/690 | 1.19(0.61-2.32) | 1.37(0.69-2.72) | 1.39(0.70-2.76) | 1.38(0.69-2.74) |
| Q4(60.7-85.3) | 13/689 | 1.28(0.63-2.61) | 1.17(0.56-2.46) | 1.21(0.58-2.53) | 1.21(0.58-2.53) |
| Q5(≥85.4) | 24/689 | 3.23(1.73-6.02) | 3.08(1.49-6.37) | 3.43(1.66-7.10) | 3.48(1.69-7.21) |
| *P* _for trend_ |  | <0.001 | 0.006 | 0.003 | 0.003 |
| **HbAA+HbGA** | |  |  |  |  |
| Q1(<76.3) | 20/688 | 1 | 1 | 1 | 1 |
| Q2(76.4-97.4) | 13/689 | 0.97(0.48-1.97) | 1.00(0.49-2.04) | 1.04(0.50-2.13) | 1.03(0.50-2.12) |
| Q3(97.6-122.9) | 14/688 | 0.88(0.41-1.86) | 0.91(0.43-1.94) | 0.92(0.43-1.96) | 0.91(0.43-1.93) |
| Q4(123.0-188.1) | 12/689 | 1.45(0.74-2.86) | 1.40(0.70-2.79) | 1.44(0.72-2.89) | 1.43(0.71-2.87) |
| Q5(>188.2) | 25/689 | 3.63(1.93-6.83) | 3.23(1.50-6.98) | 3.52(1.63-7.56) | 3.62(1.68-7.79) |
| *P* _for trend_ |  | <0.001 | 0.011 | 0.007 | 0.007 |

The levels of hemoglobin biomarkers of AA were presented as HRs (95% CIs). **^a^** Model 1 adjusted for age, sex, and race. **^b^** Model 2 additionally adjusted for smoking and drinking status, education level, income, BMI, regular exercise, energy and cotinine. **^c^** Model 3 further adjusted disease status of diabetes, hypertension and dyslipidemia. **^d^** Model 4 finally adjusted for INFLA-score. In addition, log-transformed levels of HbAA, HbGA and HbAA+HbGA were adjusted when analyzing the association between hemoglobin biomarkers of AA and cancer mortality. Q, quintile; HR, hazard ratio; CI, confidence interval.

**Supplement table 4**

Modification effect of hypertension, hyperlipidemia, and diabetes on the association of AA hemoglobin biomarkers with cancer mortality.

| **Variates** | **AA hemoglobin** | | **HR (95%CI)** | ***p* _for interaction_** |
| --- | --- | --- | --- | --- |
|  | **biomarkers** | |  |  |
| Smoke | |  |  |  |
|  | | HbAA | 1.01(0.84-1.22) | 0.232 |
|  | | HbGA | 1.21(1.02-1.44) | 0.920 |
|  | | HbAA+HbGA | 1.16(0.97-1.39) | 0.818 |
| Diabetes | |  |  |  |
|  | | HbAA | 1.10(0.92-1.30) | 0.662 |
|  | | HbGA | 1.26(1.06-1.49) | 0.273 |
|  | | HbAA+HbGA | 1.23(1.04-1.47) | 0.250 |
| Hypertension |  | |  |  |
|  | HbAA | | 1.05(0.81-1.35) | 0.783 |
|  | HbGA | | 1.15(0.90-1.49) | 0.686 |
|  | HbAA+HbGA | | 1.08(0.84-1.39) | 0.412 |
| Dyslipidemia |  | |  |  |
|  | | HbAA | 1.09(0.90-1.33) | 0.834 |
|  | | HbGA | 1.18(0.97-1.44) | 0.759 |
|  | | HbAA+HbGA | 1.17(0.96-1.42) | 0.885 |

Adjustments included age, sex (male/female), race (Mexican American, other Hispanic, non-Hispanic white, non-Hispanic black or other races), regular exercise (yes/no), body mass index (BMI), education (< 9th Grade, 9-11th Grade, High School Grade, GED or Equivalent, Some College or AA degree, College Graduate or above), income (< $20,000, $20,000-$45,000, $45,000-$75,000, $75,000-$100,000 or > $100,000), current smoker (yes/no), current drinker (yes/no), energy (kcal/d), cotinine (ng/ml), disease status of diabetes, hypertension and dyslipidemia and INFLA-score. Diabetes was defined by a self­reported diagnosis, a hemoglobin A1c level ≥6.5%, a fasting plasma glucose level ≥7.0mmol/L or receiving medications for diabetes; Hypertension was defined by a self-reported diagnosis, the systolic blood pressure ≥140mm Hg, the diastolic blood pressure ≥90mm Hg or receiving medications for hypertension; Dyslipidemia was defined by serum triglyceride ≥2.26 mmol/L, serum cholesterol ≥6.22 mmol/L, low­density lipoprotein cholesterol ≥4.14 mmol/L or receiving medications for dyslipidemia.

**Supplement table 5**

Multivariate adjusted ORs of HbAA, HbGA and HbAA+HbGA for prevalence of uterine cancer in total population.

| **AA Hemoglobin Biomarkers** | **Uterine cancer** | |
| --- | --- | --- |
|  | **Case/N** | **OR (95%CI)** |
| **HbAA(pmoL/g Hb)** | |  |
| T1(≤43.9) | 3/632 | 1 |
| T2(44.0-62.2) | 6/626 | 2.47(0.59-10.31) |
| T3(≥62.3) | 5/628 | 2.51(0.50-12.70) |
| *p* _for trend_ |  | 0.237 |
| **HbGA(pmoL/g Hb)** | |  |
| T1(≤43.1) | 4/625 | 1 |
| T2(43.2-64.7) | 3/628 | 1.06(0.23-4.91) |
| T3(≥64.8) | 7/633 | 4.26(1.04-17.54) |
| *p* _for trend_ |  | 0.055 |
| **HbAA+HbGA** | |  |
| T1(≤89.2) | 3/629 | 1 |
| T2(89.3-127.4) | 5/626 | 2.17(0.49-9.54) |
| T3(≥127.6) | 6/631 | 4.11(0.85-19.82) |
| *p* _for trend_ |  | 0.075 |

The levels of hemoglobin biomarkers of AA were presented as ORs (95% CIs). Adjustments included age, sex (male/female), race (Mexican American, other Hispanic, non-Hispanic white, non-Hispanic black or other races), regular exercise (yes/no), body mass index (BMI), education (< 9th Grade, 9-11th Grade, High School Grade, GED or Equivalent, Some College or AA degree, College Graduate or above), income (< $20,000, $20,000-$45,000, $45,000-$75,000, $75,000-$100,000 or > $100,000), current smoker (yes/no), current drinker (yes/no), energy (kcal/d), cotinine (ng/ml), disease status of diabetes, hypertension and dyslipidemia and INFLA-score. T, tertile; OR, odds ratio; CI, confidence interval.
